# RPLP1 promotes tumor metastasis and is associated with a poor prognosis in triple-negative breast cancer patients

**DOI:** 10.1186/s12935-018-0658-0

**Published:** 2018-10-25

**Authors:** Zhixian He, Qian Xu, Xi Wang, Jun Wang, Xiangming Mu, Yunhui Cai, Yangyang Qian, Weiwei Shao, Zhimin Shao

**Affiliations:** 1 Department of Breast Surgery, Shanghai Cancer Center, Fudan University, Shanghai, 200032 People’s Republic of China; 2 Department of General Surgery, Affiliated Hospital of Nantong University, Nantong, 226001 Jiangsu People’s Republic of China; 3 Department of General Surgery, The Fourth Affiliated Hospital of Nantong University, Yancheng, 224000 Jiangsu People’s Republic of China

**Keywords:** RPLP1, Triple-negative breast cancer, Epithelial-mesenchymal transition

## Abstract

**Background:**

Cancer metastasis is the major reason for cancer related deaths, and the mechanism of cancer metastasis still unclear. RPLP1, a member of a group of proteins known as ribosomal proteins, is associated with tumorigenesis and primary cell immortalization and is involved in cellular transformation. However, the expression and potential function of RPLP1 in TNBC remain unclear.

**Methods:**

The expression of RPLP1 in TNBC tissues and cell lines were detected with Real-Time PCR and western blotting. 81 cases of TNBC tissue samples and adjacent non-tumor tissue samples were tested by immunochemistry to determine the correlation between the RPLP1 expression and clinicopathological characteristics. In vitro, we determined the role and mechanistic pathways of RPLP1 in tumor metastasis in TNBC cell lines.

**Results:**

In this study, we detected high levels of RPLP1 expression in TNBC samples and cell lines. RPLP1 is upregulated in triple-negative breast cancer (TNBC) tissues and cells, and high expression levels correlate with an increased risk of recurrence and metastasis. Furthermore, high RPLP1 expression was associated with a poor prognosis and was an independent prognostic marker for TNBC. In RPLP1-induced cancer metastasis, RPLP1 may increase cancer cell invasion, which is likely the result of its effect on the cancer cell epithelial-mesenchymal transition.

**Conclusions:**

Altogether, our findings indicate RPLP1 is a poor prognostic potential biomarker and anti-metastasis candidate therapeutic target in triple-negative breast cancer.

## Background

Breast cancer is the most common cancer, and is a leading cause of death among females [1–3]. Triple-negative breast cancer (TNBC), which is defined by a lack of the estrogen receptor (ER), progesterone receptor (PR), and the human epidermal growth factor receptor 2 (HER2) receptor, is resistant to conventional hormone and anti-HER2-targeted therapies [4, 5]. Therefore, TNBC has a higher recurrence and mortality rate compared to other breast cancer subtypes [6]. Thus, investigating the mechanism of cancer development in TNBC tumors will likely be highly beneficial in monitoring tumor progression and enhancing the overall prognosis [7].

Ribosomes, the organelles that catalyze protein synthesis, consist of a small 40S subunit and a large 60S subunit [8]. Together these subunits are composed of four RNA species and approximately 80 structurally distinct ribosomal proteins [9]. In eukaryotes, the ribosomal stalk complex is comprised of a RP Large P0 (P0) subunit and two heterodimers of RP Large P1 (RPLP1) and RP Large P2 (RPLP2) [10]. RPLP1 plays an important role in the elongation step of protein synthesis [11]. The C-terminal end of RPLP1 is nearly identical to the C-terminal end of the ribosomal phosphoproteins P0 and P2, which can interact with P0 and P2 to form a pentameric complex consisting of P1 and P2 dimers and a P0 monomer [12, 13]. During central nervous system development, RPLP1 promotes embryonic fibroblast senescence-associated proliferation [14]. During flavivirus infection, RPLP1 and RPLP2 are important factors for virus translation and may represent a regulatory step for the translation of specific cellular mRNAs [15]. An increasing number of studies show RPLP1 plays essential roles in cancer development [16]. In colon cancer, RPLP1 expression was fivefold up-regulated in cancerous versus normal tissues [12]. RPLP1 expression is also elevated in gynecologic tumors, including endometrial and ovarian cancers [17]. However, the expression patterns of RPLP1 in breast cancer tissues, especially in TNBC, have not been thoroughly explored.

In our previous pre-experiment, we compared the proteome extracted from TNBC cancers with and without metastasis and found that RPLP1 was 1.6-fold more abundant in metastatic TNBC cancer versus non-metastatic cancer. In the current study, we further explored the role of RPLP1 in TNBC metastasis and determined that RPLP1 may be a novel prognostic marker for TNBC.

## Materials and methods

### Patients and tissue samples

TNBC tissues and adjacent non-tumorous tissues were obtained from 81 TNBC patients who underwent curative resection between 2006 and 2014 at the Affiliated Hospital of Nantong University. All cases were newly diagnosed female patients, who had not yet undergone surgery, radiotherapy, chemotherapy, or biological therapy. Survival data were acquired by periodic interviews with their relatives. Tissue samples were processed immediately following the surgical resection. For histological examination, all tumors and adjacent non-tumor tissues were fixed in formalin and embedded in paraffin blocks. Histological slides stained with hematoxylin and eosin were examined by three pathologists. All studies were approved by the Ethics Committee of Affiliated Hospital of Nantong University.

### Immunohistochemistry

TNBC sections were deparaffinized and rehydrated with graded ethanol, soaked in EDTA (1 mmol/L, pH 8.0), and then heated to 121 °C in an autoclave for three min to retrieve the antigen. After natural cooling and rinsing with phosphate-buffered saline (PBS, pH 7.2), 0.3% hydrogen peroxide was applied for 20 min to block endogenous peroxide activity. Thereafter, 10% goat serum was applied for 1 h at room temperature to block any nonspecific reactions. After washing with PBS (pH 7.2), the sections were incubated with a rabbit anti-RPLP1 polyclonal antibody (diluted 1:100; Abcam, ab121190, USA) for 2 h. Negative control slides were processed in parallel using a nonspecific IgG antibody (diluted 1:100; Abcam, USA) at the same concentration as the primary antibody. All sections were processed using the peroxidase-anti-peroxidase kit according to the manufacturer’s instructions (Dako, Germany).

### Immunohistochemical evaluation

All the immunostained slides were evaluated by three pathologists who were blinded to sample identity. To assess RPLP1 expression, at least five high-power fields (400×) were chosen for each specimen. More than 500 cells were counted to determine the mean percentage of positively stained cells. Staining results were scored semi-quantitatively. As previous reported [18], the percentage of positive cells was scored as follows: 0 (< 10%), 1 (10–30%), 2 (30–50%), and 3 (50–70%). The staining intensity was scored as follows: 0 (negative), 1 (moderate), 2 (positive), or 3 (strongly positive). The immunostaining score, which value ranged from 0 to 9, was calculated as the level of RPLP1 expression. For statistical analysis, 0–4 is defined as low expression, while 5–9 is defined as high expression.

### Western blotting

Cells were promptly homogenized in lysis buffer and then centrifuged at 13,000*g* for 20 min at 4 °C. The supernatant was diluted twofold in SDS loading buffer and denatured at 100 °C for 15 min. An equivalent amount of protein from each sample was loaded onto a 10% SDS-PAGE gel and then transferred to a PVDF membrane (Millipore, USA). The membranes were incubated overnight at 4 °C with the primary antibodies. The antibodies were as follows: anti-RPLP1 (1:500, ab121190, Abcam, Cambridge, MA, USA), anti-E-cadherin antibody (1:1000, ab1416, Abcam), anti-vimentin antibody (1:1000, ab92547, Abcam), anti-N-cadherin antibody (1:1000, ab18203, Abcam), anti-Snail antibody (1:1000, ab180714), and anti-β-actin (1:5000; Abcam). After washing three times with tris-buffered saline with 0.1% tween-20 (TBST) for 5 min each time, the membranes were then incubated with horseradish peroxidase-conjugated secondary human anti-mouse or anti-rabbit antibodies (1:2000; Abcam) for 2 h at room temperature. The bands were then detected using an enhanced chemiluminescence detection system (Bio-Rad, USA).

### Real-time quantitative PCR

The mRNA expression levels of RPLP1 in tissues were assessed by the Real-time quantitative PCR method. The total RNA of tissues was extracted using TRIzol^®^ reagent (Thermo Fisher Scientific, Carlsbad, CA) according to the manufacturers’ instructions. cDNA for mRNA was synthesized using a Omniscript Reverse Transcription kit (Qiagen, Valencia, CA). For detecting the mRNA level of *RPLP1*, qPCR was conducted on the Mastercycler Ep Realplex (Eppendorf 2S, Hamburg, Germany). β-actin was used as an internal control. The relative mRNA expression levels were evaluated by the 2^**−**ΔΔCt^ method [19]. The primer sequences were as follows: For RPLP1: forward, 5′-TGGCCTGGCTTGTTTGC-3′, reverse: 5′-CTCGGATTCTTCTTTCTTTGCTT-3′; For β-actin: forward, 5′-GCGTGACATTAAGGAGAAG-3′, reverse: 5′-GAAGGAAGGCTGGAAGAG-3′.

### Cell cultures

TNBC cell lines MDA-MB-231, MDA-MB-436, MDA-MB-468, MDA-MB-453, and the normal breast epithelial cell line MCF-10A were obtained from the Cell Bank of the Chinese Academy of Sciences (Shanghai, China). These TNBC cells were cultured in Leibovitz’s L-15 Medium (L15 medium, Gibco, USA) supplemented with 10% fetal bovine serum (FBS, Gibco, USA) in an incubator at 37 °C without CO_2_. The MCF-10A cells were cultured in MEGM medium (Lonza/Clonetics, USA) in an incubator at 37 °C with 5% CO_2_. All the cells were passaged every 3–5 days. At the time of cell culture, we have tested for Mycoplasma infection, and there is no mycoplasma infection in each culture.

### RPLP1 knockdown or overexpression vector construction and transfection

The human RPLP1 shRNA vector, which targets sequence 5′-CATTAAAGCAGCCGGTGTAAATGTTGAGC-3′, was subcloned into the PLKO.1 vector (Invitrogen), and the RPLP1 expression vector, which contains the RPLP1 cDNA sequence, was subcloned into the PLKI.1 vector (Invitrogen). For vector transfection, the cells were seeded the day before transfection using antibiotic-free L15 medium with 10% FBS. Transient transfection of the shRNA vectors or overexpression vector was carried out using Lipofectamine 2000 in OptiMEM media, as suggested by the manufacturer (Thermo-Fisher). Cells were incubated with the vectors and lipofectamine reagent complexes for 4 h at 37 °C. FBS was then added to the cells to achieve a final concentration of 10% in medium. Two days after transfection, puromycin (Sigma-Aldrich, USA) was added to the media at 1 μg/mL for 1 week of selection. The expression levels of the target genes were determined by Western blot analysis.

### Cell proliferation assay

The cell proliferation assay was performed with the Brdu assay kit according to the manufacturer’s protocol (Roche, Germany). Generally, cells were incubated with 100 µM Brdu labeling solution for 4 h at 37 °C. After removing the culture media, the cells were fixed, and the DNA was denatured with FixDenat solution. The anti-Brdu-POD working solution and substrate solution were then added, and the absorbances of the samples were measured by an ELISA plate reader at 370 nm.

### Colony formation assay

Cells were seeded in a 6-well plate at a density (1 × 10^3^ cells/well). After cultured for 10 days, cells were fixed with 4% paraformaldehyde and then counted after staining with crystal violet. Three independent experiments were performed.

### Cell invasion assay

For the invasion assay, cells were suspended in 500 µL serum-free media and placed in the upper compartment of the invasion chamber coated with Matrigel (BD Biosciences). The lower compartment was imbued with a complete medium as the chemoattractant. The experiment was performed in triplicate.

### Cell migration assay

A 500 μL cell suspension was placed in each insert chamber containing medium free of FBS, while the medium in the lower chamber contained 10% FBS. The cells that had migrated and attached to the lower surface of the insert were fixed with 4% formaldehyde and stained with crystal violet. After washing with PBS, the number of cells was counted randomly in five scopes under a microscope (400×).

### In vivo mouse model

In this study, all animal experiments were approved by the Animal Ethics committee. Nude mice from each group (n = 6) received a tail vein injection of 1 × 10^5^ cells per week for three consecutive weeks [20], and the number of cells injected into each tail vein each time was 1 × 10^5^. The presence of lung metastases was determined by hematoxylin and eosin (H&E) staining after 10 weeks.

### Statistical analysis

All statistical analyses were carried out using SPSS 20.0 (Statistical Product and Service Solutions, USA). The association between RPLP1 expression and clinicopathological features was computed using the Chi square (χ^2^) test. RPLP1 in TNBC cells was studied using the Spearman rank correlation test, because the data were not normally distributed. For survival analysis, the log rank test and Kaplan–Meier method were used. Multivariate analysis was performed with the Cox’s proportional hazards model. For all cases, P < 0.05 was considered statistically significant.

## Results

### The expression of RPLP1 in TNBC tumors, adjacent normal tissue, and cell lines

**Fig. 1:**
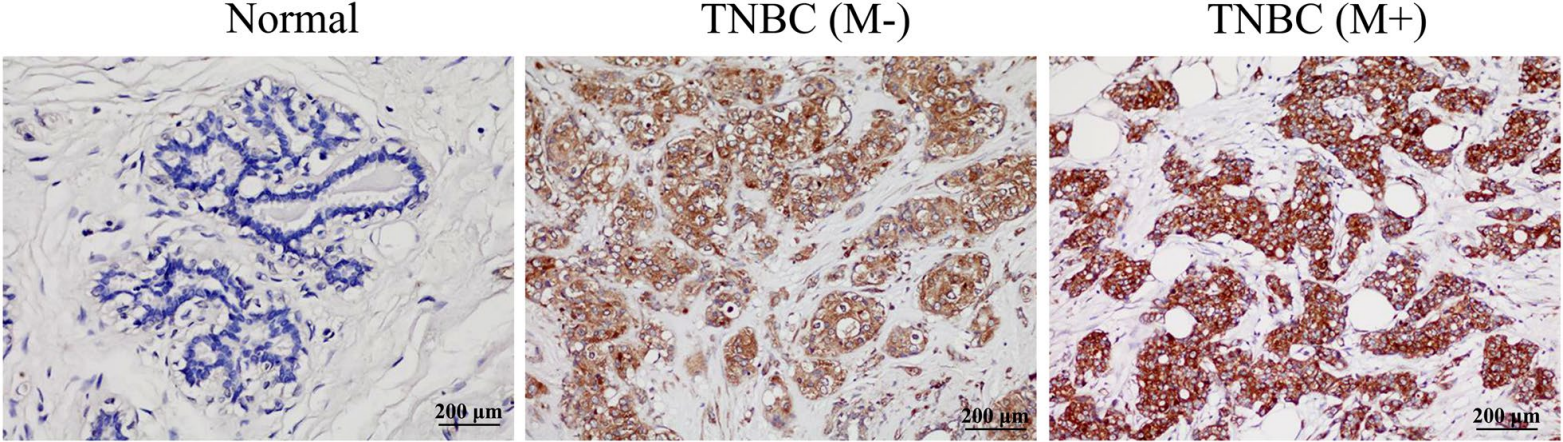
Immunohistochemical analysis of RPLP1 expression. Paraffin embedded tissue sections were stained with antibody against RPLP1, counterstained with hematoxylin, and observed under microscope (×40). Representative images are shown. Normal, normal adjacent breast tissue; TNBC(−), non-metastatic TNBC tissue; TNBC(+), metastatic TNBC tissue

**Fig. 2:**
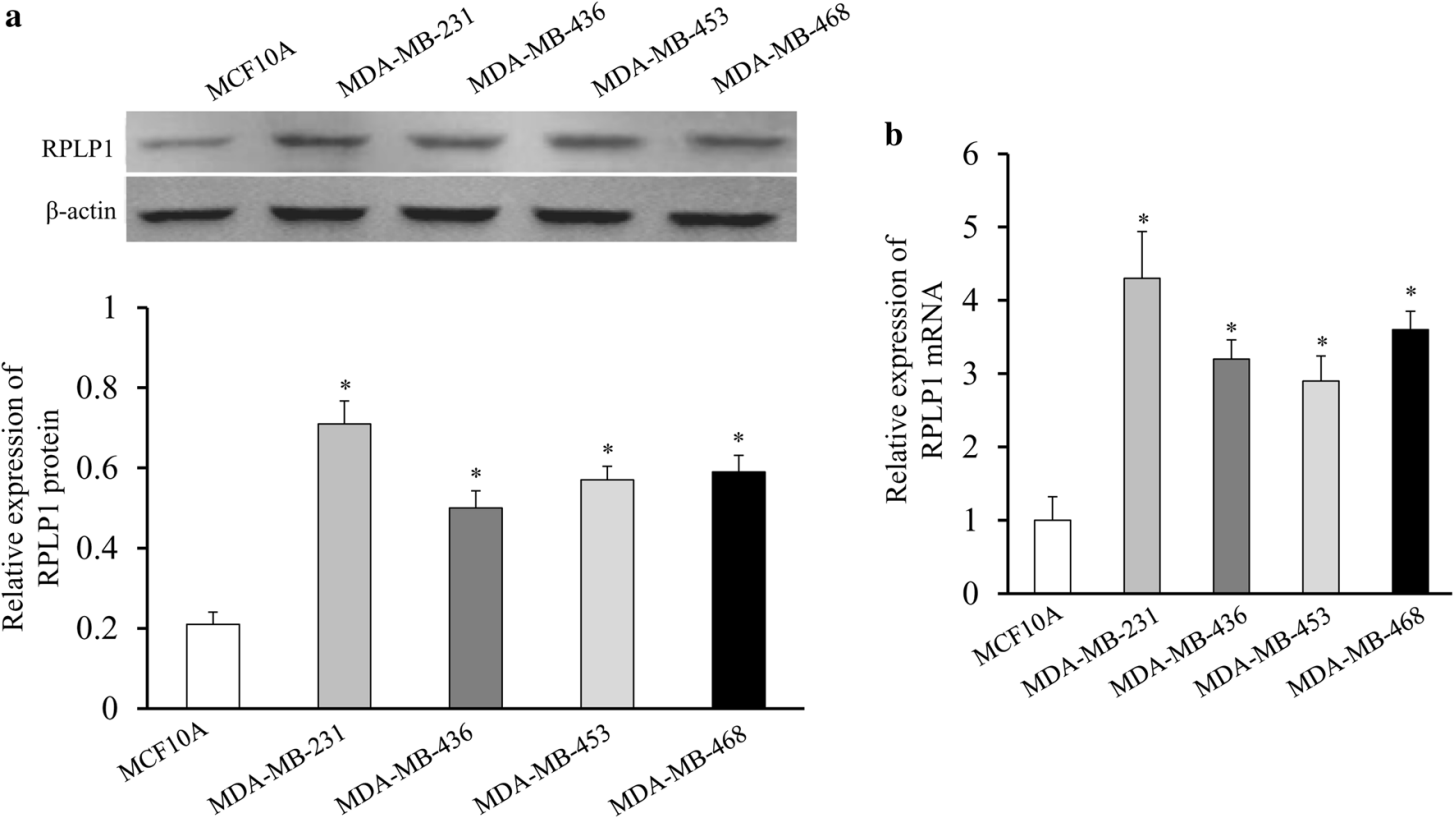
RPLP1 expression in TNBC and normal breast cells. **a** RPLP1 protein expression was higher in TNBC cells than in the normal breast cell line. The bar chart demonstrates the ratio of RPLP1 protein to β-actin using quantitative analysis. **b** RPLP1 mRNA expression was also higher in TNBC cells than in the normal breast cell line. Data are mean ± SD for three independent experiments. *P < 0.05, compared to the MCF-10A cell line

To confirm the results of our prior proteomics data, we used immunohistochemistry staining to profile the expression of RPLP1 in paraffin embedded TNBC tissues. Representative examples of reactivity for RPLP1 are shown in Fig. 1. RPLP1 expression in TNBC was scored as positive when cytoplasmic staining was strong. We demonstrated that RPLP1 expression is low in the adjacent normal tissues, while it is high in TNBC tissues. Furthermore, the expression of RPLP1 was elevated in the metastasis group compared to the metastasis negative group (Fig. 1). To confirm these immunohistochemical results, we analysed RPLP1 protein expression (via Western blotting) in TNBC and normal breast epithelial cell lines. RPLP1 protein expression was significantly higher in TNBC cells than in normal breast cells, and in MDA-MB-231 cells, the expression of RPLP1 was relatively high (Fig. 2a). Furthermore, we used quantitative PCR to verify the same expression pattern of RPLP1 in the mRNA level (Fig. 2b). These data indicate that RPLP1 expression is correlated with and may be involved in the development of TNBC.

### Correlation of RPLP1 expression with the clinicopathological parameters of TNBC

To examine the relationship between RPLP1 protein expression and common parameters associated with tumor behavior, we compared RPLP1 levels with the clinicopathological features. For statistical analysis, TNBC patients (or tumor tissue specimens) were divided into high and low RPLP1 expression groups, depending on immunostaining score. Statistical analysis indicated that RPLP1 expression was significantly associated with histologic grade (P = 0.026), axillary lymph node status (P = 0.012), and distant metastasis (P = 0.001), but there was no correlation between RPLP1 expression and other factors, such as age and tumor size (Table 1).

**Table 1:** Correlation between RPLP1 expression and the clinicopathologic features of TNBC

Clinicopathologic variables	Cases	RPLP1 level		P value	χ^2^
		Low	High		
Age	81	46	25	0.263	1.639
≤ 50	39	25	14		
> 50	42	21	21		
Tumor size (cm)	81	46	35	0.131	4.058
< 2	35	23	12		
2–5	41	22	19		
> 5	5	1	4		
Grade	81	46	35	0.026	7.308
I	22	13	9		
II	32	23	9		
III	27	10	17		
Axillary lymph node status	81	46	35	0.012	6.868
N0	48	33	15		
Nx	33	13	20		
Distant metastasis	81	46	35	0.001	12.566
Negative	60	41	19		
Positive	21	5	16		

Statistical analyses were performed by the Pearson χ^2^ test

* P < 0.05 is considered significant

### Prognostic significance of RPLP1 expression

**Fig. 3:**
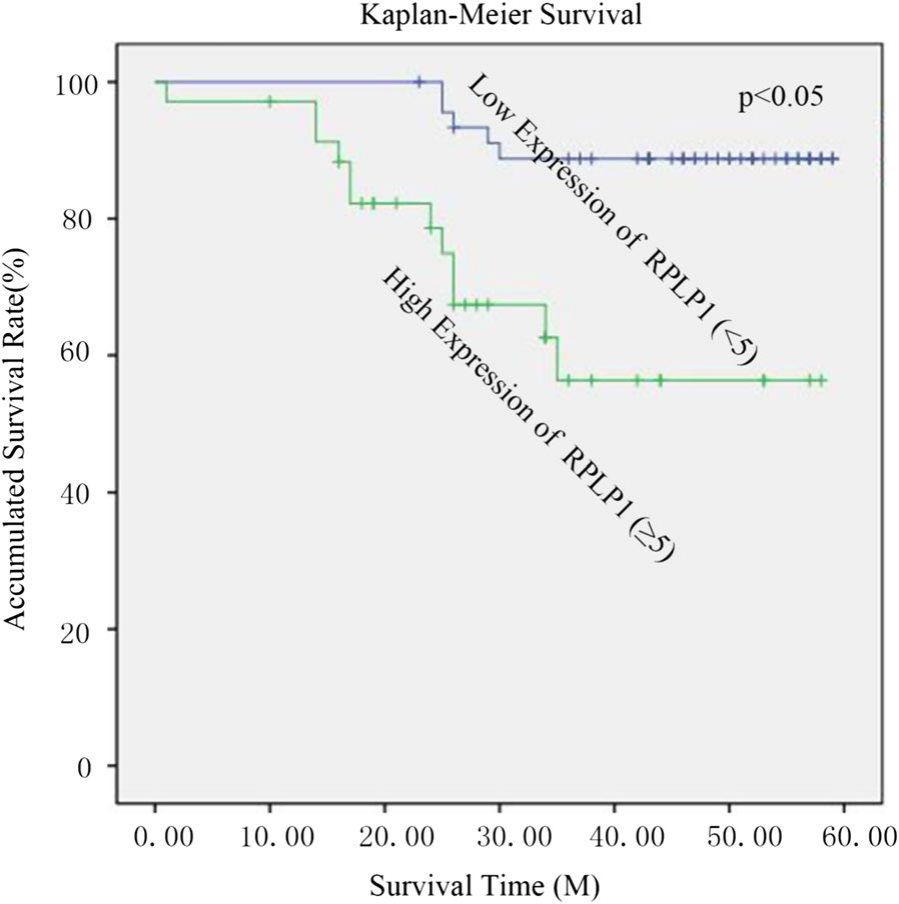
Kaplan-Meier survival curves for high versus low RPLP1 expression in 81 patients with TNBC. Patients in the high expression group had significantly shorter overall survival than those in the low expression group

Since we found that RPLP1 was associated with histologic grades, we further investigated the role of RPLP1 in breast cancer prognosis. We ultimatelydetermined that high levels of RPLP1, histologic grade, axillary lymph node status, and distant metastasis were significantly associated with patient survival states (P = 0.014, P = 0.021, P = 0.011, P = 0.034) (Table 2). Furthermore, using the *Cox’s* proportional hazards regression model, we determined that RPLP1 expression (P = 0.001), histologic grade (P = 0.002), axillary lymph node status (P = 0.001), and distant metastasis (P = 0.004) were independent prognostic factors in patients with TNBC (Table 3). In univariate analysis, using Kaplan–Meier survival curves, we also found that high RPLP1 expression was statistically correlated with poor overall survival (Fig. 3).

**Table 2:** Survival status and clinicopathological parameters in TNBC specimens

Criteria	No. case	Survival status		P value	χ^2^
		Alive	Dead		
Age					
< 50	39	34	5	0.105	3.025
≥ 50	42	30	12		
Tumor size					
≤ 2	35	27	8	0.059	5.661
2–5	41	35	6		
> 5	5	2	3		
Grade					
I	22	14	8	0.021*	7.725
II	32	30	2		
III	27	20	7		
Axillary lymph node status					
N0	48	43	5	0.011*	7.939
Nx	33	21	12		
Distant metastasis					
Negative	60	51	9	0.034*	5.003
Positive	21	13	8		
RPLP1					
Low	46	41	5	0.014*	6.572
High	35	23	12		

Statistical analyses were performed by the Pearson χ^2^ test

* P < 0.05 is considered significant

**Table 3:** Contribution of various potential prognostic factors to survival

	Hazard ratio	95% confidence interval	P
Age	1.150	0.696–1.902	0.586
Tumor size	1.472	0.887–2.443	0.135
Grade	1.890	1.267–2.819	0.002*
Axillary lymph node status	2.541	1.445–4.467	0.001*
Distant metastasis	2.526	1.337–4.770	0.004*
RPLP1	2.552	1.499–4.344	0.001*

Statistical analyses were performed by the Pearson χ^2^ test

* P < 0.05 is considered significant

### RPLP1 promotes cell invasion and migration in TNBC

**Fig. 4:**
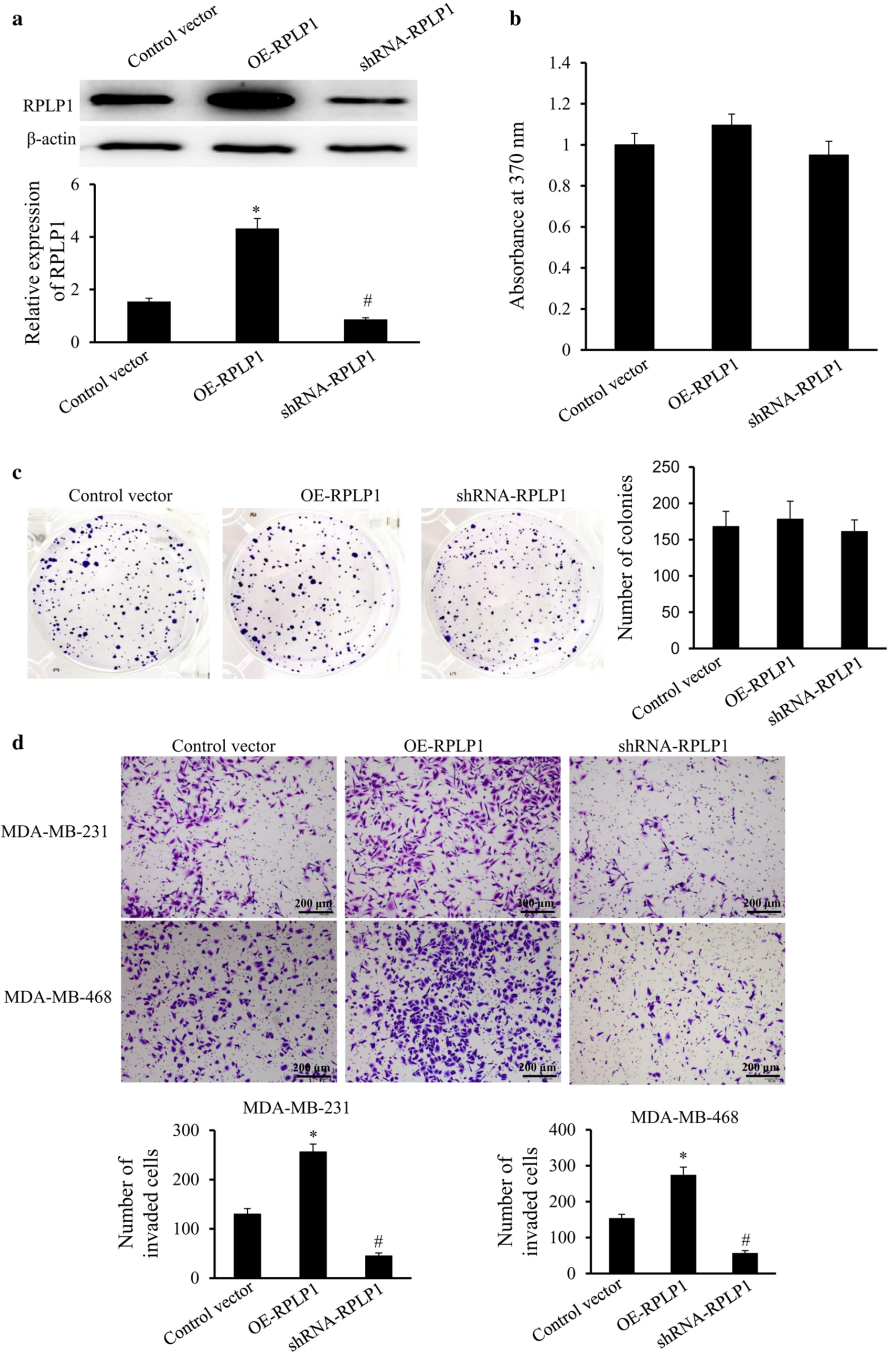
The effect of RPLP1 expression on cell behavior. **a** MDA-MB-231 cells were transfected with the control vector, RPLP1 shRNA vector, or RPLP1 expression vector. The expression of RPLP1 was detected by Western blot. **b** Brdu assays showing cell proliferation in respect to RPLP1 expression. **c** The clone formation assay shows that changes in RPLP1 expression did not affect cell clone formation abilities. **d** Matrigel transwell assay shows cell invasion when RPLP1 is overexpressed or RPLP1 expression is suppressed in MDA-MB-231 and MDA-MB-468 cells. Representative images are shown.*^,#^P < 0.05 compared to control vector

**Fig. 5:**
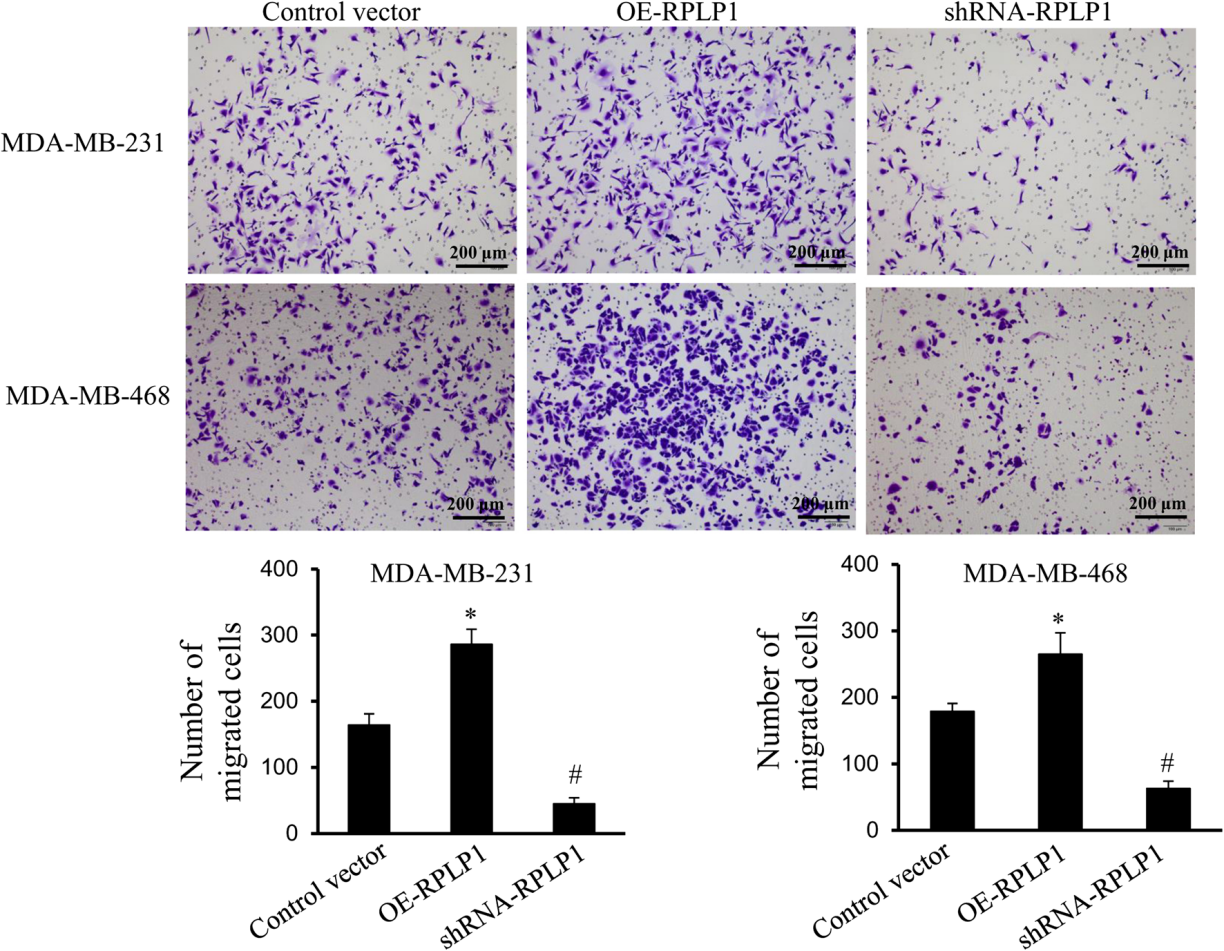
The effect of RPLP1 expression on cell migration. Transwell assay shows that inhibition of RPLP1 expression can significantly inhibit cell migration, and overexpression of RPLP1 enhanced cell migration in the MDA-MB-231 and MDA-MB-468 cell line. Representative images are shown.*^,#^P < 0.05 compared to control vector

Due to the links between RPLP1 expression and patient characteristics, we went on to investigate the role of RPLP1 in TNBC cell proliferation and invasion. When MDA-MB-231 cells were transfected with the shRNA and expression vector, we confirmed that the shRNA vector effectively knocked down RPLP1 expression, while the expression vector increased RPLP1 expression in TNBC cells (Fig. 4a). When we monitored the proliferation of these cells after 72 h of transfection, we found that changes in RPLP1 expression did not affect cell growth (Fig. 4b). In addition, the clone formation assay has been carried out and we found that changes in RPLP1 expression did not affect cell clone formation abilities (Fig. 4c). We also observe that inhibition of RPLP1 expression can significantly inhibit cell invasion, and overexpression of RPLP1 increased cell invasion in the MDA-MB-231 and MDA-MB-468 cell line (Fig. 4d). Lastly, the migration potential of cells upon overexpression and knockdown of RPLP1 has been evaluated. We also observed that inhibition of RPLP1 expression can significantly inhibit cell migration, and overexpression of RPLP1 enhanced cell migration in the MDA-MB-231 and MDA-MB-468 cell line (Fig. 5). These data indicate that RPLP1 promotes cancer metastasis in TNBC, but it may not be absolutely required for cell growth or proliferation.

### The mechanism that RPLP1 affects metastasis may be through EMT

**Fig. 6:**
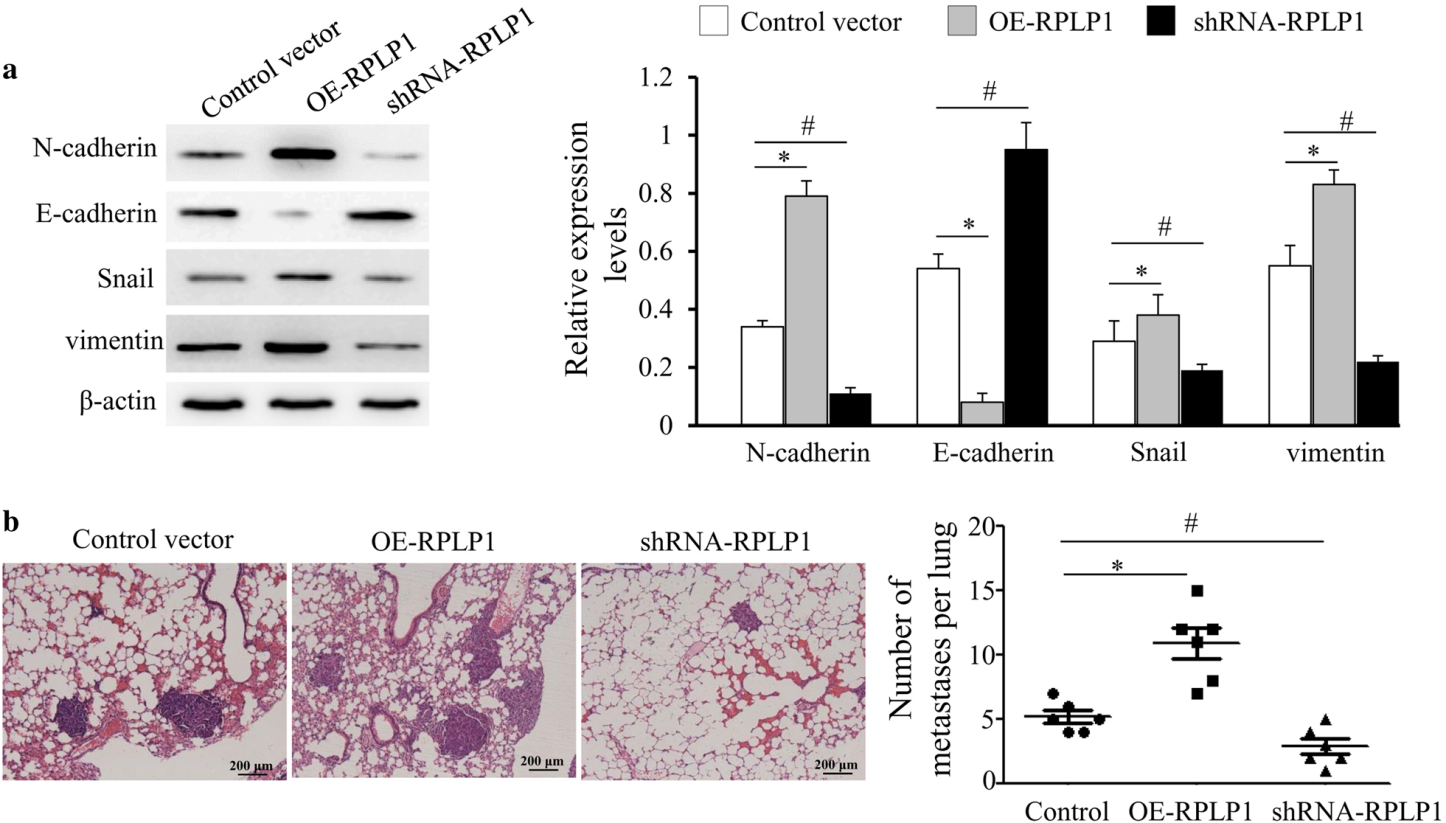
The effect of RPLP1 expression on the epithelial-mesenchymal transition. MDA-MB-231 cells were transfected with the control vector, RPLP1 shRNA vector, or RPLP1 expression vector. **a** Western blots showing the changes N-cadherin and E-cadherin expression. **b** MDA-MB-231 cells transfected with OE-RPLP1 are more metastatic than MDA-MB-231 cells transfected control vector, and cells transfected with shRNA-RPLP1 are less metastatic than MDA-MB-231 cells transfected control vector in vivo, as indicated by HE staining. Representative images are shown. *^, #^P < 0.05 compared to control vector

The epithelial-mesenchymal transition plays an essential role in TNBC metastasis. To investigate the mechanism of RPLP1 in TNBC metastasis, we examined the effect of RPLP1 on the epithelial-mesenchymal transition in MDA-MB-231 cells. When we knocked down RPLP1 expression, the epithelial cell marker E-cadherin was increased, and the mesenchymal cell marker N-cadherin was decreased. However, overexpression promoted expression of the mesenchymal cell marker N-cadherin and decreased expression of epithelial cell marker E-cadherin (Fig. 6a). These data indicate RPLP1 mediates cell invasion and affects the cell epithelial-mesenchymal transition. Histologic analysis confirmed that MDA-MB-231 overexpressed RPLP1 cells had an increased ability to metastasize to the lungs compared to the MDA-MB-231 control. Contrary, inhibition of RPLP1 expression can significantly inhibit metastasis (Fig. 6b).

## Discussion

Triple negative breast cancer bears the worst prognosis of any breast cancer, because it is resistant to most conventional therapies [21]. Metastasis is the main cause of patient death in women with TNBC. Here, we show RPLP1 expression is elevated in the TNBC, especially in metastatic tissues when compared with normal tissues, implicating RPLP1 may be involved in the development and metastasis of TNBC. In addition, we showed that RPLP1 promotes the cell epithelial-mesenchymal transition and may play a direct role in influencing cancer spread.

Other research studies have reported that RPLP1 is associated with the progression of colon cancer and gynecologic tumors [21]. In colon cancer, RPLP1 gene expression is significantly enhanced [21]. In 140 biopsies of gynecologic cancers (46 endometrioid and 94 ovarian), RPLP1 was up-regulated in 27% of the tumors [17]. As breast cancer represents another important feminine cancer, that is associated with hormone changes, we elected to investigate the role of RPLP1 in breast tumors [22]. TNBC is an especially devastating form of breast cancer that lacks expression of ER, PR, and Her-2 and therefore is generally resistant to common anti-hormone and anti-Her-2 therapies. Here, we show that RPLP1 expression is associated with TNBC and its clinicopathological features, such as axillary lymph node status, vein invasion, and metastasis. Ultimately, elevated RPLP1 expression (at both the RNA and protein level) was associated with a poor prognosis of TNBC.

When we examined the relationship between RPLP1 and tumor characteristics, we determined that high RPLP1 expression promotes cell invasion. However, knocking down RPLP1 expression did not affect cell proliferation, indicating that RPLP1 largely affects motility mechanisms and metastasis, rather than tumor growth. Increasing numbers of studies have shown that the epithelial-mesenchymal transition is a key mechanism involved in cancer metastasis [17, 23]. In conjunction with these findings, we determined that RPLP1 expression promoted the epithelial-mesenchymal transition in TNBC metastasis.

## Conclusions

This study indicated a link between RPLP1 overexpression and histologic grade, axillary lymph node status and distant metastasis in TNBC patients, which reveals RPLP1 may have significant value as a prognostic indicator for TNBC patients. Our results also indicated that RPLP1 may be a novel target to prevent or reduce breast cancer metastasis and could be the basis for future anti-cancer therapies, especially for difficult to treat TNBC.
